# Biguanide Pharmaceutical Formulations and the Applications of Bile Acid-Based Nano Delivery in Chronic Medical Conditions

**DOI:** 10.3390/ijms23020836

**Published:** 2022-01-13

**Authors:** Melissa Jones, Corina Mihaela Ionescu, Daniel Walker, Susbin Raj Wagle, Bozica Kovacevic, Jacqueline Chester, Thomas Foster, Edan Johnston, Jafri Kuthubutheen, Daniel Brown, Marcus D. Atlas, Momir Mikov, Armin Mooranian, Hani Al-Salami

**Affiliations:** 1 Biotechnology and Drug Development Research Laboratory, Curtin Medical School & Curtin Health Innovation Research Institute, Curtin University, Perth, WA 6102, Australia; melissa.a.jones@postgrad.curtin.edu.au (M.J.); c.ionescu@postgrad.curtin.edu.au (C.M.I.); daniel.walker1@postgrad.curtin.edu.au (D.W.); susbinraj.wagle@postgrad.curtin.edu.au (S.R.W.); bozica.kovacevic@postgrad.curtin.edu.au (B.K.); j.chester@student.curtin.edu.au (J.C.); thomas.p.foster@student.curtin.edu.au (T.F.); edan.johnston@student.curtin.edu.au (E.J.); 2 Hearing Therapeutics, Ear Science Institute Australia, Queen Elizabeth II Medical Centre, Perth, WA 6009, Australia; marcus.atlas@earscience.org.au; 3 Fiona Stanley Hospital, Murdoch, WA 6150, Australia; Jafri.Kuthubutheen@health.wa.gov.au; 4 Curtin Medical School, Curtin Health Innovation Research Institute, Curtin University, Perth, WA 6102, Australia; daniel.brown2@curtin.edu.au; 5 Ear Sciences Centre, The University of Western Australia, Perth, WA 6009, Australia; 6 Department of Pharmacology, Toxicology and Clinical Pharmacology, Faculty of Medicine, University of Novi Sad, 21101 Novi Sad, Serbia; momir.mikov@mf.uns.ac.rs

**Keywords:** metformin, bile acids, formulation, encapsulation, diabetes mellitus, pharmacokinetics, pharmacodynamics

## Abstract

Biguanides, particularly the widely prescribed drug metformin, have been marketed for many decades and have well-established absorption profiles. They are commonly administered via the oral route and, despite variation in oral uptake, remain commonly prescribed for diabetes mellitus, typically type 2. Studies over the last decade have focused on the design and development of advanced oral delivery dosage forms using bio nano technologies and novel drug carrier systems. Such studies have demonstrated significantly enhanced delivery and safety of biguanides using nanocapsules. Enhanced delivery and safety have widened the potential applications of biguanides not only in diabetes but also in other disorders. Hence, this review aimed to explore biguanides’ pharmacokinetics, pharmacodynamics, and pharmaceutical applications in diabetes, as well as in other disorders.

## 1. Introduction

Biguanides are a class of drugs, with one drug from this category, titled metformin, prescribed widely at present. Metformin has been marketed for several decades as a treatment for diabetes mellitus (DM) and has a well-established absorption profile. DM is a metabolic disorder and has three main, broadly characterised and often overlapping types: type 1 diabetes (T1D), type 2 diabetes (T2D), and gestational diabetes (GD) [1,2]. Loss of glucose control and haemostasis are major characteristics of DM, and inflammation remains the underpinning pathological disturbance associated with all three types of DM.

Glucose is digested and absorbed from food that contains tri, di, and mono polysaccharides, and once food is digested, glucose is released and taken up through the gastrointestinal tract into the bloodstream. Excess glucose is then stored in the liver, or converted to fat, or undergoes storage within alternative body tissues [3,4]. Haemostasis of glucose is severely disturbed in diabetic patients, although the mechanisms and causalities vary depending on the type and severity of the disease. Simplistically, T1D occurs as a result of immune-mediated destruction of insulin-producing pancreatic β-cells, resulting in partial or absolute insulin deficiency; it usually occurs in adolescence or early stages of life, but can also occur at any time, with several studies suggesting adult-onset T1D to be more prevalent than onset in children [5,6,7,8]. Whilst the onset of T1D is rather rapid, β-cell destruction itself occurs progressively, with the result being complete insulin deficiency [9,10]. The lack of insulin and subsequent increase in blood glucose levels leads to excessive breakdown of fatty acids in an attempt to meet the body’s energy needs via alternative pathways. As a result, there is an accumulation of ketones, which has the potential to cause the development of fatal ketoacidosis [11]. As a result, type 1 diabetics receive lifelong exogenous insulin injections into subcutaneous tissue or undergo delivery of exogenous insulin via an insulin pump [11,12]. With regard to T2D, the cause, pathophysiology, and short- and long-term prognosis are different than those for T1D.

**Figure 1:**
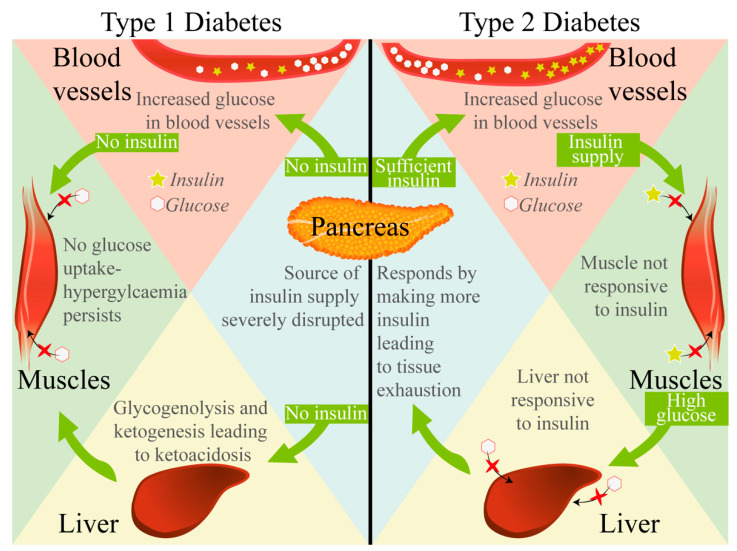
Biological differences between T1D and T2D. The differences in glucose uptake and insulin release between T1D and T2D are demonstrated in terms of the pancreas, blood vessels, muscle, and liver.

T2D is the most common type of DM, with an aetiology that is not an auto-immune disorder but rather is less understood in terms of exact causes and insulin-resistance pathophysiology. However, T2D has two common primary pathophysiologies: the inability of the pancreatic β-cells to secrete insulin appropriately, and the ineffective response of insulin-sensitive tissues to the secreted insulin. T2D results from negative interaction between genetic predisposition and environmental factors and seems to be associated with risk factors for cardiovascular disease, including dyslipidaemia, raised blood pressure, and abdominal obesity. T2D is described as defects in both insulin sensitivity and pancreatic β-cell dysfunction, with most patients manifesting the disorder as a reduced insulin response to glucose, as well as a significant resistance to endogenous insulin [12,13,14]. Although the most common prevalence of T2D is in older adults, it can also occur as early as childhood. Its prevalence in younger patients has seen a rapid increase due to multiple factors, not limited to a lack of physical activity and subsequent increasing youth obesity. Patients with this type of DM are asymptomatic initially and may not be diagnosed for several years, during which the pathophysiology continues to exacerbate and worsen [15,16]. T2D patients do not usually require exogenous insulin administration, and if they do, it is typically in late stages when endogenous insulin production is insufficient in the support of alternative treatments in overcoming the peripheral insulin resistance [16,17]. Figure 1 demonstrates the differences between T1D and T2D.

Distinguishing between T1D and T2D based on presenting features and common symptoms is not always clear and may require further clinical testing. Typically, T1D presents as an early and young-stage onset of clinical symptoms which include polydipsia, polyuria, and weight loss with ketosis. Such presentation may occur with or without a family history of autoimmune diseases. In addition to the measurement of endogenous insulin production, testing antibodies associated with T1D such as anti-GAD antibodies and anti-islet-cell antibodies can be used in the support of a diagnosis. However, antibody screening is not routinely done on patients, due to the fact that a small proportion of T1D patients do not possess positive antibodies, indicating that they do not have the required diagnostic accuracy for T1D. T1D should not be ruled out in a patient with clinical features but negative antibodies, since other co-morbidities may also exist, and these may mask the diagnosis of the disease. Genetic testing may be utilised in the diagnosis of T1D [12,18].

In contrast, T2D typically presents with obesity in addition to alternative risk factors, inclusive of family history, with a more gradual onset. A typically considered hallmark in the diagnosis of T1D is diabetic ketoacidosis, which can also occur in type 2 diabetes, albeit more rarely. Other potential non-specific physical presentations may include delayed wound healing, blurred vision, and fungal or bacterial infections [2,19].

GD is widely recognised as the most common medical condition during pregnancy, and it is described as hyperglycaemia that occurs during pregnancy, without the patient previously being diagnosed with DM. Typically, an oral glucose tolerance test is used for the diagnosis of GD. There are a multitude of potential risk factors for GD, including maternal obesity, genetic factors, and a history of GD or family history of T2D, in addition to the maternal age during pregnancy, with older ages more at risk. GD comes with an increased risk of complications, such as cardiovascular disease and T2D in the mother, and future risks of obesity, T2D, cardiovascular disease, and GD in the child [20,21,22].

GD is typically associated with both chronic insulin resistance during pregnancy and β-cell dysfunction, indicating the underlying pathophysiology to be both insulin resistance from tissues and impaired β-cell function. The insulin resistance and impaired β-cell function present in a similar manner to the hallmarks of T2D. During pregnancy, there is an increased insulin requirement, with the β-cells unable to respond in a sufficient manner and the increased metabolic stress highlighting any underlying deficiencies present from β-cells. Tissue resistance to insulin is an additional contributing factor to β-cell dysfunction, as there is a reduction in insulin-induced uptake of glucose [21,22].

DM oral treatment agents are classified into groups according to their differing mechanisms of action. These classes include insulin secretagogues, consisting of the sulphonylureas and meglitinides, a common first- or second-line therapy for T2D, which release insulin via the stimulation of pancreatic β-cells. Sulphonylureas (such as gliclazide, glibenclamide, glipizide, and glimepiride) and meglitinides (repaglinide and nateglinide) have the same mechanism of action; however, one of the key issues is that this category commonly undergoes secondary failure, losing effectiveness over time. Thiazolidinediones (rosiglitazone and pioglitazone) act by increasing the insulin sensitivity of various tissues via the promotion of glucose utilisation and decreased glucose production. However, these drugs have significant side effects, with rosiglitazone having been suspended by the European Medicines Agency. Another classification is dipeptidyl peptidase-4 inhibitors (Alogliptan, Linagliptin, Saxagliptin, Sitagliptin, and Vildaglipton), which are often used in combination therapy with metformin and insulin. This classification is considered safe with minimal adverse effects, which may include upper respiratory tract infections or headaches. One category is the sodium-glucose co-transporter 2 inhibitors (canagliflozin, empagliflozin, and Dapagliflozin), which act via the inhibition of glucose absorption renally, resulting in increased glucose excretion. These agents are associated with high levels of adverse effects, typically in the urogenital tract, with serious adverse reactions reported in the range of 1% to 12.6% of recipients. Alpha-glucosidase enzyme inhibitors (acarbose, miglitol, and voglibose) reduce the postprandial levels of triglycerides. Acarbose also possesses other benefits, such as a decreased risk of cardiovascular disease. The main side effects from this category are gastrointestinal related. Finally, there are the biguanides; metformin is a member of this class and will be discussed in detail [23,24].

## 2. Biguanides: Metformin

Metformin is an oral anti-hyperglycaemic drug and is currently the most widely used agent for the treatment of T2D [24,25]. Several associations, including the International Diabetes Federation, American Diabetes Association, and European Association for the Study of Diabetes recognise T2D as a global health challenge and recommend for the use of metformin to be commenced as the first-line treatment in all newly diagnosed patients of T2D, regardless of age or obstructive comorbidities [26,27]. Various formulations of immediate-release (IR) metformin are available including 500 mg, 850 mg, and 1000 mg tablets, in addition to extended-release (XR) formulations, also available in 500 mg, 850 mg, and 1000 mg [28,29]. Metformin was first synthesised and found to reduce blood sugar levels in the 1920s; however, it was neglected for the next two decades, due to a shift towards alternative diabetic drugs and the primary use of insulin treatments [30]. In terms of T1D, insulin is the hallmark therapy, given the absolute lack of endogenous insulin production and the fact that exogenous insulin treatment results in an otherwise normal peripheral response to the exogenous insulin’s biological actions [31].

Metformin can be investigated, analysed, and quantified in biological samples, such as plasma and tissues, through drug analytical methods using widely established calorimetry or spectrophotometry techniques. Such techniques are deployed to study both the pharmacokinetics and dose–concentration profile of the drug, as discussed in detail below [32,33,34].

Multiple techniques have been validated for the analysis of metformin. Mass spectrometry has become more favourable in the pharmaceutical industry due to its improved reproducibility and data comprehension; however, it does require a specialist skill set. Another technique involves the use of various chromatographic methods to analyse metformin, with extraction techniques such as solid phase and the derivatisation of metformin. A combined technique has become more in favour recently, with much focus on method development for chromatography–mass spectrometry analysis. This combined spectrometry includes both liquid and gas chromatography [34,35,36,37,38]. Many researchers have found that these approaches are costly and not routinely available in the clinical setting. The requirement for time-consuming chemical derivatisation reactions indicates that such methodologies are not practical for rapid, cost-effective, and, most importantly, sensitive laboratory reporting of metformin in biological samples [35]. Therefore, the majority of studies now employ high-performance liquid chromatography (HPLC), with its many benefits including simplicity, sensitivity, and rapidity. HPLC methods for the detection and quantification of metformin involve several techniques, including reverse-phase HPLC, ultrafiltration, organic solvent extractions, ion-pair exchange, and protein precipitation [35,39,40].

Various concentrations and reagents can be utilised in the creation of mobile phases, which commonly consist of acetonitrile and buffer. Of note, some groups have employed the following examples, among others: acetonitrile and phosphate buffer (65:35 *v*/*v*) adjusted with orthophosphoric acid to pH 5.75; 0.025 M ammonium sulphate and acetonitrile (7:3 *v*/*v*); ammonium formate at pH 3.0 and acetonitrile (40:60 *v*/*v*); and acetonitrile with nitric acid (30:70 *v*/*v*). Some groups have also employed methanol and a buffer, for example, 0.5 mol L^−1^ sodium hydroxide and methanol (40:60 *v*/*v*) [34,40,41].

Metformin hydrochloride, which is chemically identified as dimethylbiguanide, is an oral anti-hyperglycaemic agent that, as mentioned previously, is currently the most widely used drug within the scope of T2D treatment [42]. Metformin is the only drug from the biguanide class that is currently available on the market—a synthetically derived guanidine (*Galega officinalis*). Two other biguanides, phenformin and buformin (monosubstituted compounds that possess a long side chain) were previously removed from the market in the 1970s due to a high incidence of lactic acidosis. The higher incidence of such lactic acidosis with phenformin and buformin is believed to be due to the required therapeutic dose being high, whereby plasma lactic acid concentrations correlate to the biguanide dosage. Metformin demonstrated a better safety profile and significantly lower lactic acidosis incidence, with cases typically due to inappropriate usage or existing kidney conditions. In plasma, phenformin has a half-life of 7 to 15 h, buformin’s half-life is 4 to 6 h, whereas metformin’s half-life is 1.5 h. In terms of renal elimination, phenformin often undergoes hepatic metabolism and is renally cleared at 42–262 mL min^−1^. Buformin and metformin are excreted largely unchanged, with clearances of 393 and 440 mL min^−1^, respectively. In contrast to the commercially unavailable treatment phenformin, metformin does not possess sufficient binding to mitochondrial membranes. In addition, metformin does not inhibit the electron transport chain, which indicates it to be substantially less toxic to cells at therapeutic concentrations. Based upon these indications, metformin has been selected as the biguanide of choice in current pharmacotherapy of T2D [30,43,44].

**Figure 2:**
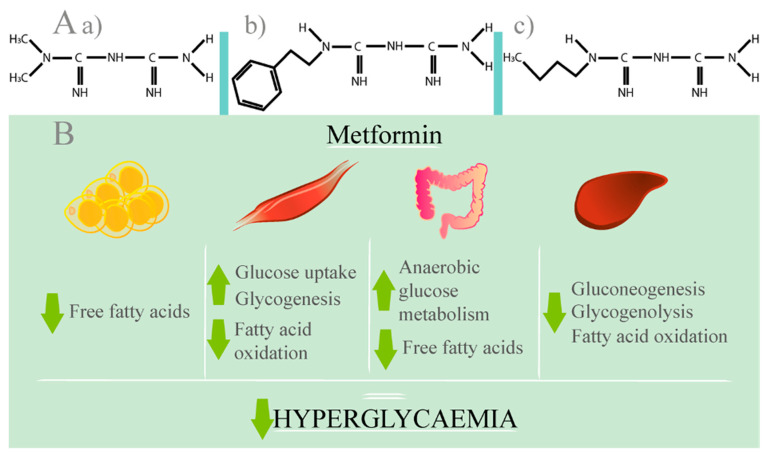
Biguanides’ structure and the historically accepted actions of metformin. Panel (**A**) illustrates the chemical structure of biguanides, including metformin; (**a**) Chemical structure of metformin; (**b**) Chemical structure of phenformin; (**c**) Chemical structure of buformin. Panel (**B**) demonstrates many historically accepted mechanisms by which metformin reduces hyperglycaemia.

Chemically speaking, biguanides are composed of two guanidine groups joined together with the loss of an ammonia. The anti-hyperglycaemic effects of biguanides are as a result of this chemical structure [45]. The rapid passive dissolution of metformin is indicated to be unlikely due to the fact that the lipid solubility of the unionised species is −1.43, indicating a low log P value. In comparison to the log P of −0.84 from phenformin, metformin’s partition coefficient is lower. This is as a result of the two methyl substituents of its structure, which impart less lipophilicity than the larger phenylethyl side chain that phenformin possesses, as seen in Figure 2 (panel A) [42].

In the literature, metformin’s dosage is reported as a hydrochloride salt (MW 165.63); in contrast, concentrations in biological fluids are expressed as the free base (MW 129.16). Despite the fact that in the last 50 years metformin has been regarded as one of the most effective therapeutics used to treat T2D, the underlying molecular and cellular mechanisms of action are still largely unexplored and debated [45]. Early models suggested that increased metformin accumulation in skeletal muscle resulted in an increased glucose uptake. However, these models present an unlikely mechanism due to the high therapeutic dose of metformin required for this effect. One widely accepted model for the glucose-lowering effect is the enhancement of hepatic sensitivity to insulin, causing a reduction in hepatic glucose output, which is achieved via gluconeogenesis inhibition as a result of mitochondrial inhibitors. Despite the mechanism of action of metformin in hepatocytes remaining uncertain, it is thought that metformin’s primary site of action is in the mitochondria. Consequently, there is an increase in peripheral insulin sensitivity. The preferential effects of metformin action in the liver, rather than skeletal muscles, may be due to the fact that the supply of the drug is direct from the gut via the portal vein. This indicates that metformin reaches the liver in higher concentrations than what is seen in the other peripheral organs or tissues. Furthermore, there are higher levels of Organic Cationic Transporter 1 (OCT1) that actively transport metformin to the hepatocytes where its action site is located. Hence, metformin does not alter insulin production itself; instead, tissue sensitivity to the available insulin content is enhanced. The wide therapeutic window of metformin and—in comparison to other drugs for T2D treatment—the low risk of hypoglycaemia is due to this unique mechanism of action. In addition, there is no weight gain associated, nor is there cardiovascular risk [45,46,47,48]. Panel B of Figure 2 demonstrates some of the potential mechanisms of metformin.

Furthermore, studies have demonstrated metformin’s ability to increase glucagon-like peptide 1 (GLP-1). GLP-1 is secreted via endocrine L cells, which are present throughout the intestine, most prominently in the ileum and colon. Routinely, GLP-1 occurs due to nutrients, resulting in increased insulin via glucose induction whilst inhibiting the secretion of glucagon. In addition, increasing GLP-1 results in a reduced appetite due to the delayed emptying of the gastric system. Studies have suggested that metformin has the capacity to increase this GLP-1 secretion, thus indicating that metformin may also have impacts in the intestines, as well as in the liver [49,50].

With the intention of a constant rate of release of metformin along the gastrointestinal tract in mind, continuous SR, also known as controlled release (CR), formulations of metformin have been developed. SR formulations have one key disadvantage in that the overwhelming majority of the drug undergoes deposition in locations where metformin’s absorption is very low [51]. As a result of such a disadvantage, the literature has debated the rationality of SR formulations’ availability in the market in comparison to XR formulations [28,29]. Thus, the high water solubility and incomplete absorption of metformin within the gastrointestinal tract have necessitated research into novel oral delivery platforms that could optimise the drug release. The aim of such an optimised platform would allow release within the optimal absorption site, which would result in targeted delivery, as well as the potential to offer a controlled manner of release [52]. For several reasons, including those aforementioned, microencapsulation of metformin with the use of polymers is gaining rapid momentum; this is discussed further below.

## 3. Microencapsulation

A microcapsule is defined as a spherical particle, typically within the size range of 1 μm to 1000 μm, consisting of a distinctive core and a different distinctive surrounding material. Microencapsulation, the technique of creating microcapsules, may be utilised in the food, cosmetics, and pharmaceutical industries. The aim in pharmaceutics is typically in the development of drug delivery systems, to improve the delivery of pre-existing drugs, and to allow novel medications to become available. Microcapsules serve many purposes, primarily in the protection of a sensitive core material from a hostile external environment whilst maintaining its therapeutic capabilities. In terms of drug delivery, it is the process whereby drugs are entrapped within a polymer matrix in order to not only control the site of their delivery along the gastrointestinal tract, but to also allow for a constant rate of drug release. Further benefits to such an industry may include decreased side effects, longer shelf life, and allowing oral routes of ingestion, subsequently increasing patient compliance. Microcapsules must be safe and biocompatible, as well as possessing consistent and predictable diffusion and degradation kinetics [53,54,55,56,57,58,59].

There are many microencapsulation techniques, broadly categorised as physical, chemical, and physicochemical, which often overlap. The creation of microcapsules is achieved via the use of specific formulatory combinations of various polymers and hydrogels that can produce a delivery system offering unique physicochemical properties. As a consequence, desirable drug release profiles can be achieved via such a technique [53,54,55,56,60,61,62,63]. One favoured method of microcapsule formation is the ionic gelation method as it avoids the use of the toxic organic solvents required in solvent evaporation techniques. In addition, the high temperatures that are seen in spray drying methodologies are not required, and the use of complex instrumentation is avoided. Other key benefits of the ionic gelation methodology are the short processing time requirements and the high encapsulation efficiencies that are achieved. Furthermore, the physicochemical properties of the resulting microcapsules can be influenced and manipulated positively via the encapsulation parameters [54,64,65,66,67,68,69]. As briefly mentioned, one important criterion is the use of ideal encapsulation biomaterials and polymers, which will be discussed in detail.

## 4. Nanoencapsulation

Nanocapsules are defined as having a size range of 10 nm to 1000 nm; however, this range is often debated [60]. Given the size and nature of nanocapsules, they offer the potential for precise drug targeting, specificity, and improved bioavailability in comparison to their microcapsule counterparts. Similar to microcapsules, nanocapsules consist of a central core, typically of the active material, and a surrounding material, typically consisting of polymers or other excipients [70,71,72].

There are several methodologies for the creation of nanocapsules, with many of these adjusted and developed based upon existing microencapsulation techniques. However, these are often more complex than microencapsulation and require selection based upon the ideal properties of the resultant nanocapsules. Electrospraying and nanospray drying techniques have both demonstrated the ability to produce nanocapsules, with electrospraying resulting in smaller, more consistent capsules. Freeze drying alone does not produce nanocapsules, although it may be utilised to stabilise nanocapsules whilst retaining their nanoscale. Coacervation techniques may also be applied to form nanocapsules, modified from microencapsulation. However, this technique requires the optimisation of conditions to a narrow range, with these conditions differing depending upon what is being encapsulated [70,73,74]. Nanoemulsions may also be utilised and have shown some promise in the pharmaceutical industry. They may be utilised in the delivery of lipophilic substances and can assist in the delivery of both hydrophilic and hydrophobic drugs, forming nanocapsules with size ranges below 200 nm [75,76]. To improve the structure, stability, and biocompatibility, added excipients are often utilised, as discussed further below.

## 5. Potential Encapsulation Materials

In terms of encapsulation, different materials, components, or excipients are utilised in the creation of nano- or microcapsules. These are selected based upon their properties and the desired properties of the resultant capsules. They may be synthetic, semi-synthetic, or natural, with sodium alginate (SA), a natural polymer, and several synthetic polymers being some of the most commonly used. These materials are imperative to the pharmacokinetics of the resultant drug delivery system, whilst themselves not producing therapeutic actions [57,61,62,63,77,78,79,80]. SA is one of the most common natural polymers used in encapsulation and has been utilised in both nano- and microencapsulation. It is considered as nontoxic, biocompatible, and biodegradable, with the ability to be utilised in the controlled release of drugs for a pH-sensitive delivery system [68,81,82,83]. Some synthetic polymers commonly utilised include polyethers, primarily polyethylene glycol (PEG), which is water soluble and nontoxic, and polyesters, including poly (lactic-co-glycolic acid) (PLGA), which is considered highly biocompatible. Poloxamers, which are thermosensitive, have also been investigated as excipients in drug delivery [84,85].

Bile acids (BA)s are natural, amphiphilic molecules found in humans which have an important role in fat absorption. BAs have been utilised in encapsulation for the delivery of various drugs, primarily due to their solubilising ability and potential to improve bioavailability, including that of non-polar drugs [2,69,86,87]. Attention has also recently been given to BAs in the field of diabetes. This is as a result of the glucose-lowering effect of BAs, as well as their permeation-enhancing effect in vitro, ex vivo, and in vivo [2,88,89,90,91]. It has been hypothesised that the delivery of BAs to the distal gastrointestinal tract may in fact enhance their potential glucose-lowering effects and, therefore, the management of T2D [50].

## 6. Metformin–Bile Acid Interaction

**Figure 3:**
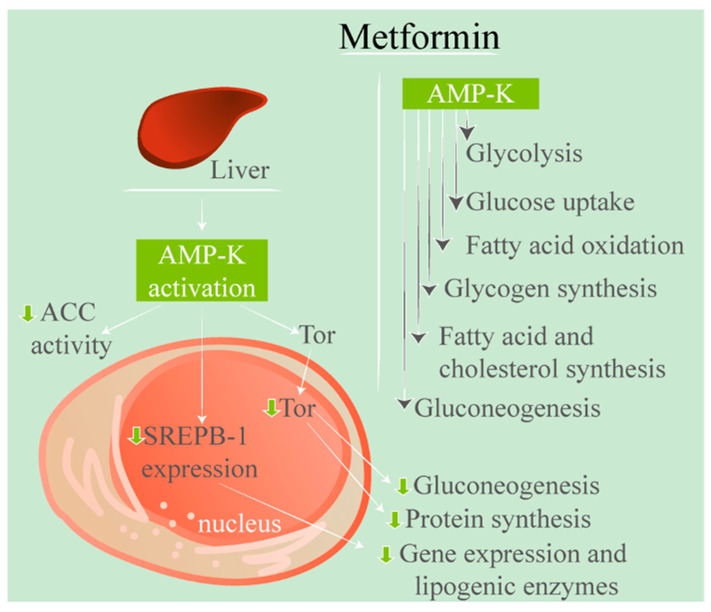
Representation of metformin’s effects from AMP-K activation. As can be seen, metformin acts upon the liver to activate AMP-K, which results in a cascade effect.

As previously mentioned, metformin has an impact on GLP-1; however, the exact mechanism is debated. Despite this debate, GLP-1 is recognised as being stimulated by BAs [49,92,93]. Metformin has been shown to impact the farnesoid X receptor, inhibiting it via a mechanism mediated by AMP-K which causes a decrease of BA absorption in the ileum, increasing the BA concentration. This increase can result in stimulation of GLP-1. Metformin activates AMP-K, hence metformin’s impact on the BAs in the intestines and subsequent glucose-lowering effects [49,93,94]. This higher intestinal BA concentration due to inhibited resorption may also explain some of the side effects of metformin in the gastrointestinal system, including diarrhoea due to increased BA excretion [50]. Figure 3 demonstrates this AMP-K activation.

Some BAs such as chenodeoxycholic acid have been demonstrated in studies to inhibit the rate-limiting step in cholesterol synthesis via hydroxymethylglutaryl-coenzyme A (HMG-CoA). This property indicates it for use in the management of hypertriglyceridaemia, rheumatoid arthritis, congenital liver disease, and constipation [95]. Metformin was investigated in the context of a preliminary study assessing any changes in oral–cecal transit and whether the drug had the ability to result in malabsorption of bile salts. The study was conducted via the use of a lactulose breath test and orally administered 14C-glycocholate followed by breath 14CO_2_ measurement over 6 h, as well as stool collection for 72 h, respectively. The rationale for the study was the examination of metformin and the resultant effects on the physiology of the gut, with specific focus drawn on the causation of such changes. Furthermore, an investigation was conducted on the negative impacts seen in the lower gastrointestinal tract of some patients on metformin treatment, and whether these are as a result of one or several effects, including variation in bacterial colonisation of the small bowel due to alteration of intestinal transit or interruption of bile salt enterohepatic circulation. Such interruption of the bile salt enterohepatic circulation was observed via the increased breath 14CO_2_ and 14C faecal excretion. Thus, the results gave the conclusion that malabsorption of bile salts occurs in the ileum [96]. Furthermore, metformin was found to increase faecal bile salt excretion, with more oral malabsorption when compared to parental metformin as illustrated via in vivo studies [97]. Contrasting results were reported by another group, with this research team investigating metformin’s impacts on bile salt metabolism. In their study, 14C-glycocholate testing was used and showed that 4 days of treatment with metformin gave rise to a 5-fold increase in BA deconjugation, but in contrast to the aforementioned study, these researchers described a decrease in faecal BA excretion within their patient population of type 2 diabetics [98].

## 7. Encapsulation of Metformin

**Figure 4:**
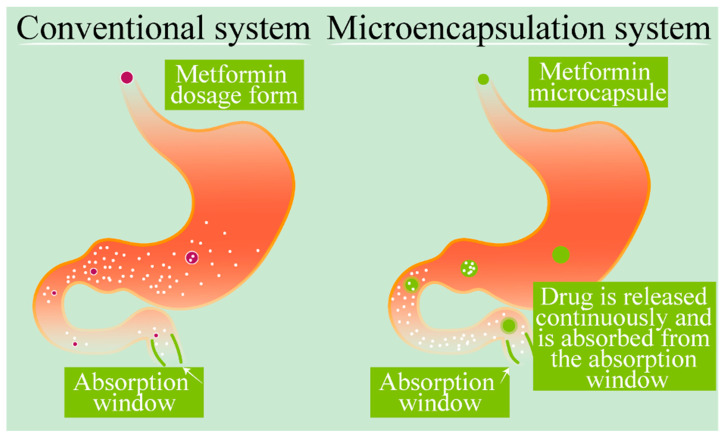
Metformin delivery: conventional versus microencapsulation. Demonstration of a traditional metformin dosage form showing large drug release prior to the absorption window. In comparison, microencapsulation of metformin retains the drug to allow a continuous release to the absorption window.

In terms of metformin, a controlled release system needs to be developed that would allow the retention of the drug in the gastrointestinal tract long enough to release a constant amount of metformin within its absorbance location, the small intestine. Currently, metformin’s limitations are primarily due to its low efficiency in oral delivery, which also limits its potential uses as a treatment for other disease types aside from diabetes. One technique that may assist in overcoming these known limitations is the nano- or microencapsulation of metformin. The preparation techniques deployed commonly for such encapsulation include solvent evaporation, utilising ethylcellulose and Eudragits, and spray drying and ionic gelation utilising alginate/polymer combinations [51,64,99,100,101,102]. Figure 4 presents a traditional metformin dosage system in comparison to a microencapsulated delivery system. Metformin nanotechnological approaches have also been investigated; however, more research is required to find an ideal, long-term, biocompatible, and cost-effective solution. Nanotechnology also offers the potential to utilise metformin’s properties as a treatment for other metabolic disorders and cancers, indicating the value of this ongoing research [103].

Preclinical studies investigating the pharmacokinetics and pharmacodynamics of metformin micro- and nanoparticles made via such methods have shown promising results. Such results have included long-term improvements in blood glucose levels, as well as enhancing the oral bioavailability of metformin as compared to that of the conventional formulations [51]. Based upon these preliminary studies, there is potential for the microencapsulation of metformin to be used for the enhancement of oral dosing for human diabetic patients. However, further studies and, most importantly, thorough clinical studies must be conducted in order to develop a definitive conclusion about the safety and effectiveness of microencapsulated metformin.

Shivhare et al. prepared metformin microcapsules with the polymers cellulose acetate, ethylcellulose, and Eudragit S100 via an oil-in-oil solvent evaporation technique [104]. Mancer et al. produced metformin microcapsules via a double emulsion process and complex coacervation. They utilised the polymers soy protein isolate and pectin, resulting in the easily reproduceable production of microcapsules with promising characteristics, including size and size distribution [102]. Shehata et al. used a nanospray dryer to form nanocapsules of metformin, SA, and gelatin. Their studies indicate the potential of nanotechnology to allow sustained and controlled release of metformin [105]. Therefore, nano- and microencapsulation of metformin may be utilised to improve delivery not only for diabetes treatments, but for the treatment of alternative disorders, too.

## 8. Metformin as an Alternative Treatment

Whilst metformin still remains the drug of choice for many in the treatment of T2D, its properties indicate its strong potential for the treatment of other metabolic disorders; however, as previously mentioned, its variability in oral delivery restricts these therapeutic benefits [103]. Key properties from metformin that may be exploited for alternative disease treatments include improved haemostasis, oxidative stress, endothelial function regulation, the redistribution of fat and lipid profiles, and a new focus looking at its antioxidant properties. Some studies also suggest anti-proliferative effects, as well as potential neuroprotection in cancer research [106,107,108].

In addition to the treatment of T2D, metformin has also been assessed for use in the prevention of T2D or the delay of onset for those at risk of disease development. Studies have been conducted utilising placebo groups, diet and exercise groups, and groups on metformin. Overall, whilst diet and exercise were the most effective, metformin still significantly reduced, by 31%, the risk of T2D development in comparison to the placebo group [106,108,109]. This potentially offers a significant advantage in the delay or prevention of the onset of T2D; however, more research is required to understand more about the mechanisms by which metformin has caused these positive results. Furthermore, metformin has been found to reduce the risk of cardiovascular events in patients with T2D, with more research required to understand the mechanism of action. Metformin has demonstrated anti-atherosclerotic effects, assisting in this reduction in both incidence and mortality associated with cardiovascular events, with the potential to be more widely translated as a treatment for non-diabetic patients [110,111]. Kulkarni et al. demonstrated metformin’s potential effects on ageing, both metabolically and non-metabolically, indicating potential new pathways and usages of metformin, particularly those which are age-associated [112].

Metformin’s antioxidant properties are not fully understood; however, these properties indicate its potential as a treatment for various conditions that occur as a result of oxidative stress. Such properties may also assist in explaining the results of studies into metformin’s antiproliferative and anti-tumoral effects on a range of cancers. Metformin’s reduction in the risk of lifetime cancer development in diabetic patients has previously been demonstrated to be a 25 to 30% reduction. The mechanism of action behind these results is not understood; however, similar trials utilising other anti-diabetic drugs did not demonstrate such results, indicating that the anti-diabetic effects are not the explanation [106,108,113,114].

## 9. Hearing Loss

Hearing loss has a major impact on people’s lives, changing the way they communicate, and is associated with large costs, both medically and non-medically. The World Health Organisation estimates that 5% of the global population suffer from disabling hearing loss. Hearing loss has long been associated with diabetes, with increased risk demonstrated by many in comparison to the general population, strengthening evidence that hearing loss may be a comorbidity of diabetes [115,116,117].

Several studies have investigated the potential for the utilisation of metformin in the treatment of hearing loss. Much of this has come from the demonstration that metformin can reduce oxidative stress and has a neuroprotective effect. Oishi et al. found that metformin was protective against hair cell death in vitro; however, it was not protective against gentamicin-induced ototoxicity in guinea pigs in vivo. One possible explanation for such findings may be the route of metformin delivery. They delivered metformin via subcutaneous injection, which may not have been effective due to metformin’s hydrophilic nature [118,119]. Whilst the study offered a potentially advantageous use of metformin in vitro, it was unable to be replicated in vivo. However, an alternative delivery method, such as the use of nanocapsules, may assist in averting the hydrophilic nature of metformin. Chen et al. investigated whether a link exists between metformin usage and the risk of sudden sensorineural hearing loss in diabetic patients. Their large-scale study had two groups of diabetic patients, one on metformin treatment and one not. Their hearing was measured over a period of 14 years; those who were taking metformin had a lower incidence of sudden sensorineural hearing loss than did those who were not. This indicates a potential link between reducing diabetic hearing loss and metformin; however, the study did not provide any research into the mechanism by which metformin may assist in this prevention, due to its population-based style [120]. Hence, whilst showing promising results, further research is required on this topic to investigate the causative relationship of this potential link.

Gedik et al. conducted studies on rats and hearing loss based upon the literature indicating that metformin possesses antioxidant properties [121]. Similarly, Kesici et al. investigated whether metformin is effective in the protection against noise-induced hearing loss in rats. In their study, they used four groups of rats, including controls. The metformin was dissolved in saline and dosed to the rats via gavage. Noise exposure was conducted via white noise subjection. Overall, their study demonstrated that metformin was preventative of a permanent threshold shift in the hearing levels [122]. Whilst these results are promising, they may be improved via the nano- or microencapsulation of metformin, with the process potentially overcoming any disadvantageous properties of metformin, such as its hydrophilic nature.

## 10. Limitations and Future Perspectives

Much of the research covered throughout this review comprises small-scale studies that have not been further examined. In addition, many results have not been investigated to understand the mechanisms behind them. Therefore, to have truly advantageous studies, additional research is required to investigate the specific mechanisms and gain an understanding of metformin’s impacts in the aforementioned studies, which have demonstrated promising results in various medical conditions, particularly diabetes and its associated conditions, in addition to hearing loss.

As discussed, more research is required to understand all the properties of metformin and how the mechanisms of action provide the discussed clinical effects. With this understanding, metformin may be able to be further utilised in the treatment of not only metabolic but also additional, alternative disorders. Whilst some research has been conducted in these areas, further studies are required to explain the results of many of the published studies and to understand how metformin acted to produce the published results.

Furthermore, metformin may be able to be further utilised if it can be encapsulated, via either nano- or microencapsulation, in order to improve its bioavailability and offer controlled, sustained release. Based upon the reviewed literature, further studies would need to be conducted in order to find the ideal formulation of this encapsulation. A commercially viable production technique for the creation of both nano- and microcapsules further widens the scope of production in all areas, not only for metformin. In addition, the encapsulation of metformin opens possibilities in which it may be utilised in the treatment of a wide array of disorders.

## 11. Conclusions

Overall, of the category of biguanides, metformin is the common, marketable drug. Metformin has many properties that have not been thoroughly explored, with a studied safety profile. These properties offer the potential to be manipulated for the treatment of not only diabetes, for which it is commonly prescribed, but other disorders, including metabolic disorders and hearing loss. Nano- and microencapsulation are continually emerging technologies that offer an alternative delivery system. These technologies offer enhanced distribution that may be utilised in metformin delivery, thus allowing metformin to be available for the treatment of more than just the type 2 diabetes for which it is regularly prescribed. In conclusion, metformin shows much promise in the treatment of a wide range of disorders, which may be improved via the utilisation of encapsulation technologies.

## References

[1] Wang G.S., Hoyte C. (2018). Review of biguanide (metformin) toxicity. J. Intensive Care Med..

[2] Negrulj R., Mooranian A., Al-Salami H. (2013). Potentials and limitations of bile acids in type 2 diabetes mellitus: Applications of microencapsulation as a novel oral delivery system. J. Endocrinol. Diabetes Mellit..

[3] Gromova L., Fetissov S., Gruzdkov A. (2021). Mechanisms of glucose absorption in the small intestine in health and metabolic diseases and their role in appetite regulation. Nutrients.

[4] Adeva-Andany M.M., Pérez-Felpete N., Fernández-Fernández C., Donapetry-García C., Pazos-García C. (2016). Liver glucose metabolism in humans. Biosci. Rep..

[5] Eizirik D.L., Colli M.L., Ortis F. (2009). The role of inflammation in insulitis and β-cell loss in type 1 diabetes. Nat. Rev. Endocrinol..

[6] Di Meglio L.A., Evans-Molina C., Oram R.A. (2018). Type 1 diabetes. Lancet.

[7] Diaz-Valencia P.A., Bougnères P., Valleron A.-J. (2015). Global epidemiology of type 1 diabetes in young adults and adults: A systematic review. BMC Public Health.

[8] Leslie R.D., Evans-Molina C., Freund-Brown J., Buzzetti R., Dabelea D., Gillespie K.M., Goland R., Jones A.G., Kacher M., Phillips L.S. (2021). Adult-onset type 1 diabetes: Current understanding and challenges. Diabetes Care.

[9] Burrack A.L., Martinov T., Fife B.T. (2017). T Cell-mediated beta cell destruction: Autoimmunity and alloimmunity in the context of type 1 diabetes. Front. Endocrinol..

[10] Chen C., Cohrs C.M., Stertmann J., Bozsak R., Speier S. (2017). Human beta cell mass and function in diabetes: Recent advances in knowledge and technologies to understand disease pathogenesis. Mol. Metab..

[11] Inzucchi S.E. (2012). Diagnosis of diabetes. N. Engl. J. Med..

[12] Jenkins A.J., O’Neal D.N., Nolan C.J., Januszewski A.S., Hardikar A. (2016). The pathobiology of diabetes mellitus. Pancreatic Islet Biology.

[13] Henriksen E.J., Diamond-Stanic M.K., Marchionne E.M. (2011). Oxidative stress and the etiology of insulin resistance and type 2 diabetes. Free Radic. Biol. Med..

[14] Galicia-Garcia U., Benito-Vicente A., Jebari S., Larrea-Sebal A., Siddiqi H., Uribe K.B., Ostolaza H., Martín C. (2020). Pathophysiology of type 2 diabetes mellitus. Int. J. Mol. Sci..

[15] Jensen E.T., Dabelea D. (2018). Type 2 Diabetes in youth: New lessons from the SEARCH study. Curr. Diabetes Rep..

[16] Vajravelu M.E., Lee J.M. (2018). Identifying prediabetes and type 2 diabetes in asymptomatic youth: Should HbA1c be used as a diagnostic approach?. Curr. Diabetes Rep..

[17] Lebovitz H.E. (2011). Insulin: Potential negative consequences of early routine use in patients with type 2 diabetes. Diabetes Care.

[18] Punthakee Z., Goldenberg R., Katz P. (2018). Definition, classification and diagnosis of diabetes, prediabetes and metabolic syndrome. Can. J. Diabetes.

[19] Goyal R., Jialal I. (2020). Diabetes Mellitus Type 2.

[20] McIntyre H.D., Catalano P., Zhang C., Desoye G., Mathiesen E.R., Damm P. (2019). Gestational diabetes mellitus. Nat. Rev. Dis. Primer..

[21] Plows J.F., Stanley J.L., Baker P.N., Reynolds C.M., Vickers M.H. (2018). The pathophysiology of gestational diabetes mellitus. Int. J. Mol. Sci..

[22] Johns E.C., Denison F.C., Norman J.E., Reynolds R.M. (2018). Gestational diabetes mellitus: Mechanisms, treatment, and complications. Trends Endocrinol. Metab..

[23] Phung O.J., Scholle J.M., Talwar M., Coleman C. (2010). Effect of noninsulin antidiabetic drugs added to metformin therapy on glycemic control, weight gain, and hypoglycemia in type 2 diabetes. JAMA.

[24] Marín-Peñalver J.J., Martín-Timón I., Sevillano-Collantes C., Del Cañizo-Gómez F.J. (2016). Update on the treatment of type 2 diabetes mellitus. World J. Diabetes.

[25] Cho K., Chung J.Y., Cho S.K., Shin H.-W., Jang I.-J., Park J.-W., Yu K.-S., Cho J.-Y. (2015). Antihyperglycemic mechanism of metformin occurs via the AMPK/LXRα/POMC pathway. Sci. Rep..

[26] Nathan D.M., Buse J.B., Davidson M.B., Heine R.J., Holman R.R., Sherwin R., Zinman B. (2006). Management of hyperglycemia in type 2 diabetes: A consensus algorithm for the initiation and adjustment of therapy a consensus statement from the American Diabetes Association and the European Association for the study of diabetes. Diabetes Care.

[27] IDF CGTF (2006). Global guideline for type 2 diabetes: Recommendations for standard, comprehensive, and minimal care. Diabet. Med. J. Br. Diabet. Assoc..

[28] Derosa G., D’Angelo A., Romano D., Maffioli P. (2017). Effects of metformin extended release compared to immediate release formula on glycemic control and glycemic variability in patients with type 2 diabetes. Drug Des. Dev. Ther..

[29] Ali S., Fonseca V. (2012). Overview of metformin: Special focus on metformin extended release. Expert Opin. Pharmacother..

[30] Bailey C.J. (2017). Metformin: Historical overview. Diabetologia.

[31] Haller M.J., Atkinson M.A., Schatz D. (2005). Type 1 Diabetes mellitus: Etiology, presentation, and management. Pediatric Clin..

[32] Kajbaf F., Bennis Y., Hurtel-Lemaire A.-S., Andréjak M., Lalau J.-D. (2015). Unexpectedly long half-life of metformin elimination in cases of metformin accumulation. Diabet. Med..

[33] Jagdale S., Patil S., Kuchekar B., Chabukswar A. (2011). Preparation and characterization of metformin hydrochloride-compritol 888 ATO solid dispersion. J. Young Pharm..

[34] Hussain I., Ali I., Rahman H., Ghani S. (2017). Novel contribution of chromatography in the development and analyses of metformin hydrochloride in biological and environmental samples. J. Liq. Chromatogr. Relat. Technol..

[35] Porta V., Schramm S.G., Kano E.K., Koono E.E., Armando Y.P., Fukuda K., Serra C.H.D.R. (2008). HPLC-UV determination of metformin in human plasma for application in pharmacokinetics and bioequivalence studies. J. Pharm. Biomed. Anal..

[36] Zhang X., Peng Y., Wan P., Yin L., Wang G., Sun J. (2013). Simultaneous determination and pharmacokinetic study of metformin and pioglitazone in dog plasma by LC-MS-MS. J. Chromatogr. Sci..

[37] Buchberger A.R., Delaney K., Johnson J., Jillian J. (2017). Mass spectrometry imaging: A review of emerging advancements and future insights. Anal. Chem..

[38] Nespor B., Andrianova A., Pollack S., Pfau C., Arifuzzaman M., Islam N., Kubátová A., Hossain K. (2020). Metformin uptake and translocation in chickpeas: Determination using liquid chromatography–mass spectrometry. ACS Omega.

[39] Amini H., Ahmadiani A., Gazerani P. (2005). Determination of metformin in human plasma by high-performance liquid chromatography. J. Chromatogr. B.

[40] Kar M., Choudhury P. (2009). HPLC method for estimation of metformin hydrochloride in formulated microspheres and tablet dosage form. Indian J. Pharm. Sci..

[41] Nal A. (2009). Spectrophotometric and HPLC determinations of anti-diabetic drugs, rosiglitazone maleate and metformin hydrochloride, in pure form and in pharmaceutical preparations. Eur. J. Med. Chem..

[42] Graham G.G., Punt J., Arora M., Day R., Doogue M.P., Duong J.K., Furlong T.J., Greenfield J.R., Greenup L.C., Kirkpatrick C. (2011). Clinical pharmacokinetics of metformin. Clin. Pharmacokinet..

[43] DeFronzo R., Fleming G.A., Chen K., Bicsak T.A. (2016). Metformin-associated lactic acidosis: Current perspectives on causes and risk. Metabolism.

[44] Vaman Rao C., Wexler P. (2005). Biguanides. Encyclopedia of Toxicology.

[45] Rena G., Pearson E.R., Sakamoto K. (2013). Molecular mechanism of action of metformin: Old or new insights?. Diabetologia.

[46] Emami Riedmaier A., Fisel P., Nies A.T., Schaeffeler E., Schwab M. (2013). Metformin and cancer: From the old medicine cabinet to pharmacological pitfalls and prospects. Trends Pharmacol. Sci..

[47] Song R. (2016). Mechanism of metformin: A tale of two sites. Diabetes Care.

[48] Foretz M., Guigas B., Bertrand L., Pollak M., Viollet B. (2014). Metformin: From mechanisms of action to therapies. Cell Metab..

[49] Bahne E., Sun E.W.L., Young R.L., Hansen M., Sonne D.P., Hansen J.S., Rohde U., Liou A.P., Jackson M.L., De Fontgalland D. (2018). Metformin-induced glucagon-like peptide-1 secretion contributes to the actions of metformin in type 2 diabetes. JCI Insight.

[50] Sansome D.J., Xie C., Veedfald S., Horowitz M., Rayner C.K., Wu T. (2019). Mechanism of glucose-lowering by metformin in type 2 diabetes: Role of bile acids. Diabetes Obes. Metab..

[51] Pandit V., Pai R.S., Yadav V., Devi K., Surekha B.B., Inamdar M.N., Suresh S. (2012). Pharmacokinetic and pharmacodynamic evaluation of floating microspheres of metformin hydrochloride. Drug Dev. Ind. Pharm..

[52] Choudhury P.K., Kar M. (2008). Controlled release metformin hydrochloride microspheres of ethyl cellulose prepared by different methods and study on the polymer affected parameters. J. Microencapsul..

[53] Narayana Reddy C.L., Swamy B.Y., Prasad C., Madhusudhan Rao K., Prabhakar M., Aswini C., Subha M.C.S., Chowdoji Rao K. (2012). Development and characterization of chitosan-poly (vinyl pyrrolidone) blend microspheres for controlled release of metformin hydrochloride. Int. J. Polym. Mater..

[54] Whelehan M., Marison I.W. (2011). Microencapsulation using vibrating technology. J. Microencapsul..

[55] Jones M., Walker D., Ionescu C.M., Kovacevic B., Wagle S.R., Mooranian A., Brown D., Al-Salami H. (2020). Microencapsulation of Coenzyme Q10 and bile acids using ionic gelation vibrational jet flow technology for oral delivery. Ther. Deliv..

[56] Singh M., Hemant K., Ram M., Shivakumar H. (2010). Microencapsulation: A promising technique for controlled drug delivery. Res. Pharm. Sci..

[57] Mooranian A., Zamani N., Takechi R., Luna G., Mikov M., Goločorbin-Kon S., Kovacevic B., Arfuso F., Al-Salami H. (2020). Modulatory nano/micro effects of diabetes development on pharmacology of primary and secondary bile acids concentrations. Curr. Diabetes Rev..

[58] Mooranian A., Negrulj R., Al-Salami H., Morahan G., Jamieson E. (2016). Designing anti-diabetic β-cells microcapsules using polystyrenic sulfonate, polyallylamine, and a tertiary bile acid: Morphology, bioenergetics, and cytokine analysis. Biotechnol. Prog..

[59] Al-Salami H., Mooranian A., Negrulj R., Chen-Tan N., Al-Sallami H.S., Fang Z., Mukkur T., Mikov M., Goločorbin-Kon S., Watts G.F. (2014). Microencapsulation as a novel delivery method for the potential antidiabetic drug, Probucol. Drug Des. Dev. Ther..

[60] Paulo F., Santos L. (2017). Design of experiments for microencapsulation applications: A review. Mater. Sci. Eng. C.

[61] Wagle S.R., Kovacevic B., Walker D., Ionescu C.M., Jones M., Stojanovic G., Kojic S., Mooranian A., Al-Salami H. (2020). Pharmacological and advanced cell respiration effects, enhanced by toxic human-bile nano-pharmaceuticals of probucol cell-targeting formulations. Pharmaceutics.

[62] Mooranian A., Zamani N., Takechi R., Al-Sallami H., Mikov M., Goločorbin-Kon S., Kovacevic B., Arfuso F., Al-Salami H. (2019). Probucol-poly(meth)acrylate-bile acid nanoparticles increase IL-10, and primary bile acids in prediabetic mice. Ther. Deliv..

[63] Mamo J.C., Lam V., Al-Salami H., Brook E., Mooranian A., Nesbit M., Graneri L., D’Alonzo Z., Fimognari N., Stephenson A. (2018). Sodium alginate capsulation increased brain delivery of probucol and suppressed neuroinflammation and neurodegeneration. Ther. Deliv..

[64] Kulkarni N.B., Wakte P.S., Naik J.B. (2013). Metformin hydrochloride microparticles for oral controlled release: Effect of formulation variables. Int. J. Pharm. Pharm. Sci..

[65] Çetin M., Atila A., Sahin S., Vural I. (2011). Preparation and characterization of metformin hydrochloride loaded-Eudragit®RSPO and Eudragit®RSPO/PLGA nanoparticles. Pharm. Dev. Technol..

[66] Balasubramaniam J., Rao V.U., Vasudha M., Babu J., Rajinikanth P. (2007). Sodium alginate microspheres of metformin HCl: Formulation and in vitro evaluation. Curr. Drug Deliv..

[67] Nayak A.K., Pal D., Pradhan J., Hasnain M.S. (2013). Fenugreek seed mucilage-alginate mucoadhesive beads of metformin HCl: Design, optimization and evaluation. Int. J. Biol. Macromol..

[68] Mooranian A., Negrulj R., Mathavan S., Martinez J., Sciarretta J., Chen-Tan N., Mukkur T.K., Mikov M., Lalic-Popovic M., Stojancevic M. (2015). An advanced microencapsulated system: A platform for optimized oral delivery of antidiabetic drug-bile acid formulations. Pharm. Dev. Technol..

[69] Mooranian A., Negrulj R., Mathavan S., Martinez J., Sciarretta J., Chen-Tan N., Mukkur T., Mikov M., Lalic-Popovic M., Stojančević M. (2014). Stability and release kinetics of an advanced gliclazide-cholic acid formulation: The use of artificial-cell microencapsulation in slow release targeted oral delivery of antidiabetics. J. Pharm. Innov..

[70] Saifullah M., Shishir M.R.I., Ferdowsi R., Tanver Rahman M.R., Van Vuong Q. (2019). Micro and nano encapsulation, retention and controlled release of flavor and aroma compounds: A critical review. Trends Food Sci. Technol..

[71] Katouzian I., Jafari S.M. (2016). Nano-encapsulation as a promising approach for targeted delivery and controlled release of vitamins. Trends Food Sci. Technol..

[72] Patra J.K., Das G., Fraceto L.F., Campos E.V.R., del Pilar Rodriguez-Torres M., Acosta-Torres L.S., Diaz-Torres L.A., Grillo R., Swamy M.K., Sharma S. (2018). Nano based drug delivery systems: Recent developments and future prospects. J. Nanobiotechnol..

[73] Pérez-Masiá R., López-Nicolás R., Periago M.J., Ros G., Lagaron J.M., López-Rubio A. (2015). Encapsulation of folic acid in food hydrocolloids through nanospray drying and electrospraying for nutraceutical applications. Food Chem..

[74] Shishir M.R.I., Xie L., Sun C., Zheng X., Chen W. (2018). Advances in micro and nano-encapsulation of bioactive compounds using biopolymer and lipid-based transporters. Trends Food Sci. Technol..

[75] Simonazzi A., Cid A.G., Villegas M., Romero A.I., Palma S.D., Bermúdez J.M., Grumezescu A.M. (2018). Chapter 3—Nanotechnology applications in drug controlled release. Drug Targeting and Stimuli Sensitive Drug Delivery Systems.

[76] Patel R.B., Patel M.R., Thakore S.D., Patel B.G., Grumezescu A.M. (2017). Chapter 17–Nanoemulsion as a valuable nanostructure platform for pharmaceutical drug delivery. Nano- and Microscale Drug Delivery Systems.

[77] Bruschi M.L. (2015). Drug delivery systems. Strategies to Modify the Drug Release from Pharmaceutical Systems.

[78] Tønnesen H.H., Karlsen J. (2002). Alginate in drug delivery systems. Drug Dev. Ind. Pharm..

[79] Wagle S.R., Kovacevic B., Walker D., Ionescu C.M., Shah U., Stojanovic G., Kojic S., Mooranian A., Al-Salami H. (2020). Alginate-based drug oral targeting using bio-micro/nano encapsulation technologies. Exp. Opin. Drug Deliv..

[80] Mooranian A., Jones M., Ionescu C., Walker D., Wagle S., Kovacevic B., Chester J., Foster T., Johnston E., Mikov M. (2021). Advancements in assessments of bio-tissue engineering and viable cell delivery matrices using bile acid-based pharmacological biotechnologies. Nanomaterials.

[81] Szekalska M., Puciłowska A., Szymańska E., Ciosek P., Winnicka K. (2016). Alginate: Current use and future perspectives in pharmaceutical and biomedical applications. Int. J. Polym. Sci..

[82] Chaturvedi K., Ganguly K., More U.A., Reddy K.R., Dugge T., Naik B., Aminabhavi T.M., Noovli M.N., Hasnain M.S., Nayak A.K. (2019). Chapter 3—Sodium alginate in drug delivery and biomedical areas. Natural Polysaccharides in Drug Delivery and Biomedical Applications.

[83] Ahmad Raus R., Wan Nawawi W.M.F., Nasaruddin R.R. (2020). Alginate and alginate composites for biomedical applications. Asian J. Pharm. Sci..

[84] Hamid Akash M.S., Rehman K., Chen S. (2015). Natural and synthetic polymers as drug carriers for delivery of therapeutic proteins. Polym. Rev..

[85] Bodratti A.M., Alexandridis P. (2018). Formulation of poloxamers for drug delivery. J. Funct. Biomater..

[86] Pavlović N., Goločorbin-Kon S., Đanić M., Stanimirov B., Al-Salami H., Stankov K., Mikov M. (2018). Bile acids and their derivatives as potential modifiers of drug release and pharmacokinetic profiles. Front. Pharmacol..

[87] Mooranian A., Ionescu C.M., Wagle S.R., Kovacevic B., Walker D., Jones M., Chester J., Johnson E., Danic M., Mikov M. (2021). Chenodeoxycholic acid pharmacology in biotechnology and transplantable pharmaceutical applications for tissue delivery: An acute preclinical study. Cells.

[88] Negrulj R., Mooranian A., Chen-Tan N., Al-Sallami H.S., Mikov M., Golocorbin-Kon S., Fakhoury M., Watts G., Arfuso F., Al-Salami H. (2015). Swelling, mechanical strength, and release properties of probucol microcapsules with and without a bile acid, and their potential oral delivery in diabetes. Artif. Cells Nanomed. Biotechnol..

[89] Mooranian A., Negrulj R., Al-Sallami H.S., Fang Z., Mikov M., Golocorbin-Kon S., Fakhoury M., Arfuso F., Al-Salami H. (2014). Release and swelling studies of an innovative antidiabetic-bile acid microencapsulated formulation, as a novel targeted therapy for diabetes treatment. J. Microencapsul..

[90] Mooranian A., Negrulj R., Al-Sallami H.S., Fang Z., Mikov M., Golocorbin-Kon S., Fakhoury M., Watts G., Matthews V., Arfuso F. (2014). Probucol release from novel multicompartmental microcapsules for the oral targeted delivery in type 2 diabetes. AAPS PharmSciTech.

[91] Staels B., Kuipers F. (2007). Bile acid sequestrants and the treatment of type 2 diabetes mellitus. Drugs.

[92] Brighton C., Rievaj J., Kuhre R.E., Glass L., Schoonjans K., Holst J.J., Gribble F., Reimann F. (2015). Bile acids trigger GLP-1 release predominantly by accessing basolaterally located G protein–coupled bile acid receptors. Endocrinology.

[93] Kuhre R.E., Albrechtsen N.W., Larsen O., Jepsen S.L., Balk-Møller E., Andersen D., Deacon C., Schoonjans K., Reimann F., Gribble F. (2018). Bile acids are important direct and indirect regulators of the secretion of appetite- and metabolism-regulating hormones from the gut and pancreas. Mol. Metab..

[94] McCreight L.J., Bailey C.J., Pearson E.R. (2016). Metformin and the gastrointestinal tract. Diabetologia.

[95] Broughton G. (1994). Chenodeoxycholate: The bile acid. The drug. A review. Am. J. Med. Sci..

[96] Scarpello J., Hodgson E., Howlett H. (1998). Effect of metformin on bile salt circulation and intestinal motility in type 2 diabetes mellitus. Diabet. Med..

[97] Tomkin G.H. (1976). Comparison of the effect of parenteral with oral biguanide therapy on vitamin B12 and bile acid absorption. Ir. J. Med. Sci..

[98] Caspary W.F., Zavada I., Reimold W., Deuticke U., Emrich D., Willms B. (1977). Alteration of bile acid metabolism and vitamin-B12-absorption in diabetics on biguanides. Diabetologia.

[99] Mazumder B., Bera K., Sarwa K.K. (2013). Metformin HCl loaded mucoadhesive agar microspheres for sustained release. Asian J. Pharm..

[100] Snima K., Jayakumar R., Unnikrishnan A., Nair S.V., Lakshmanan V.-K. (2012). O-Carboxymethyl chitosan nanoparticles for metformin delivery to pancreatic cancer cells. Carbohydr. Polym..

[101] Bhattacharya A. (2009). Release kinetics of metformin hydrochloride microencapsulated in Isabgol husk and sagu starch hydrophilic matrix. Indian Drugs.

[102] Mancer D., Allemann E., Daoud K. (2018). Metformin hydrochloride microencapsulation by complex coacervation: Study of size distribution and encapsulation yield using response surface methodology. J. Drug Deliv. Sci. Technol..

[103] Chen Y., Shan X., Luo C., He Z. (2020). Emerging nanoparticulate drug delivery systemsof metformin. J. Pharm. Investig..

[104] Shivhare U., Darakh V., Mathur V., Bhusari K., Godbole M. (2009). Preparation and evaluation of metformin hydrochloride microcapsules. Res. J. Pharm. Technol..

[105] Shehata T.M., Ibrahima M.M. (2019). BÜCHI nano spray dryer B-90: A promising technology for the production of metformin hydrochloride-loaded alginate–gelatin nanoparticles. Drug Dev. Ind. Pharm..

[106] Rojas L.B.A., Gomes M.B. (2013). Metformin: An old but still the best treatment for type 2 diabetes. Diabetol. Metab. Syndr..

[107] Kinaan M., Ding H., Triggle C.R. (2015). Metformin: An old drug for the treatment of diabetes but a new drug for the protection of the endothelium. Med. Princ. Pr..

[108] Nasri H., Rafieian-Kopaei M. (2014). Metformin: Current knowledge. J. Res. Med. Sci..

[109] Knowler W.C., Barrett-Connor E., Fowler S.E., Hamman R.F., Lachin J.M., Walker E.A., Nathan D.M., Diabetes Prevention Program Research Group (2002). Reduction in the incidence of type 2 diabetes with lifestyle intervention or metformin. N. Engl. J. Med..

[110] Luo F., Das A., Chen J., Wu P., Li X., Fang Z. (2019). Metformin in patients with and without diabetes: A paradigm shift in cardiovascular disease management. Cardiovasc. Diabetol..

[111] Zhang K., Yang W., Dai H., Deng Z. (2020). Cardiovascular risk following metformin treatment in patients with type 2 diabetes mellitus: Results from meta-analysis. Diabetes Res. Clin. Pr..

[112] Kulkarni A.S., Brutsaert E.F., Anghel V., Zhang K., Bloomgarden N., Pollak M., Mar J.C., Hawkins M., Crandall J.P., Barzilai N. (2018). Metformin regulates metabolic and nonmetabolic pathways in skeletal muscle and subcutaneous adipose tissues of older adults. Aging Cell.

[113] Leone A., Di Gennaro E., Bruzzese F., Avallone A., Budillon A. (2014). New Perspective for an Old Antidiabetic Drug: Metformin as Anticancer Agent.

[114] Ben Sahra I., Le Marchand-Brustel Y., Tanti J.-F., Bost F. (2010). Metformin in cancer therapy: A new perspective for an old antidiabetic drug?. Mol. Cancer Ther..

[115] Kim M.-B., Zhang Y., Chang Y., Ryu S., Choi Y., Kwon M.-J., Moon I.J., Deal J.A., Lin F.R., Guallar E. (2017). Diabetes mellitus and the incidence of hearing loss: A cohort study. Int. J. Epidemiol..

[116] Helzner E.P., Contrera K.J. (2015). Type 2 diabetes and hearing impairment. Curr. Diabetes Rep..

[117] Hlayisi V.-G., Petersen L., Ramma L. (2018). High prevalence of disabling hearing loss in young to middle-aged adults with diabetes. Int. J. Diabetes Dev. Ctries..

[118] Oishi N., Kendall A., Schacht J. (2014). Metformin protects against gentamicin-induced hair cell death in vitro but not ototoxicity in vivo. Neurosci. Lett..

[119] Baldassari S., Solari A., Zuccari G., Drava G., Pastorino S., Fucile C., Marini V., Daga A., Pattarozzi A., Ratto A. (2018). Development of an injectable slow-release metformin formulation and evaluation of its potential antitumor effects. Sci. Rep..

[120] Chen H.-C., Chung C.-H., Lu C.-H., Chien W.-C. (2019). Metformin decreases the risk of sudden sensorineural hearing loss in patients with diabetes mellitus: A 14-year follow-up study. Diabetes Vasc. Dis. Res..

[121] Gedik Ö., Doğan R., Babademez M.A., Karataş E., Aydin M.S., Koçyiğit A., Eşrefoğlu M., Özturan O. (2020). Therapeutic effects of metformin for noise induced hearing loss. Am. J. Otolaryngol..

[122] Kesici G.G., Öcal F.C.A., Gürgen S.G., Erdem Ş.R., Öğüş E., Erbek H.S., Özlüoğlu L.N. (2018). The protective effect of metformin against the noise-induced hearing loss. Eur. Arch. Otorhinolaryngol..

